# LINC01638 sustains human mesenchymal stem cell self-renewal and competency for osteogenic cell fate

**DOI:** 10.1038/s41598-023-46202-z

**Published:** 2023-11-20

**Authors:** Jonathan A. R. Gordon, Coralee E. Tye, Bodhisattwa Banerjee, Prachi N. Ghule, Andre J. van Wijnen, Fleur S. Kabala, Natalie A. Page, Michelle M. Falcone, Janet L. Stein, Gary S. Stein, Jane B. Lian

**Affiliations:** 1 Department of Biochemistry, University of Vermont, Burlington, VT 05405 USA; 2 University of Vermont Cancer Center, Larner College of Medicine, University of Vermont, 89 Beaumont Avenue, Burlington, VT 05405 USA

**Keywords:** Biochemistry, RNA, Cell biology, Cell growth, Senescence

## Abstract

The skeleton forms from multipotent human mesenchymal stem cells (hMSCs) competent to commit to specific lineages. Long noncoding RNAs (lncRNAs) have been identified as key epigenetic regulators of tissue development. However, regulation of osteogenesis by lncRNAs as mediators of commitment to the bone phenotype is largely unexplored. We focused on LINC01638, which is highly expressed in hMSCs and has been studied in cancers, but not in regulating osteogenesis. We demonstrated that LINC01638 promotes initiation of the osteoblast phenotype. Our findings reveal that LINC01638 is present at low levels during the induction of osteoblast differentiation. CRISPRi knockdown of LINC01638 in MSCs prevents osteogenesis and alkaline phosphatase expression, inhibiting osteoblast differentiation. This resulted in decreased MSC growth rate, accompanied by double-strand breaks, DNA damage, and cell senescence. Transcriptome profiling of control and LINC01638-depleted hMSCs identified > 2000 differentially expressed mRNAs related to cell cycle, cell division, spindle formation, DNA repair, and osteogenesis. Using ChIRP-qPCR, molecular mechanisms of chromatin interactions revealed the LINC01638 locus (Chr 22) includes many lncRNAs and bone-related genes. These novel findings identify the obligatory role for LINC01638 to sustain MSC pluripotency regulating osteoblast commitment and growth, as well as for physiological remodeling of bone tissue.

## Introduction

Long noncoding RNAs (lncRNAs) are recognized as essential epigenetic mediators of gene expression to establish cell phenotypes and support tissue development from stem cells^1–3^. Several lncRNAs have been associated with bone formation^4,5^. The interplay between MSCs and osteoblasts with lncRNAs and their associated miRNAs has been explored^6^. LINC01638 has been studied in cancers that metastasize to bone^7^. Here we addressed LINC01638 regulation of MSC differentiation to the osteoblast lineage^8^. LncRNAs are functional noncoding RNAs that are present at low levels in most cells^9^. Numerous studies have confirmed their roles in transcriptional, post-transcriptional, and epigenetic gene regulation. These transcripts are reported to play major roles in a broad range of human diseases through several distinct molecular mechanisms^10^. Of significant importance are the chromatin interactions mediated by lncRNAs, as this greatly impacts the lncRNAs detection of the regulatory elements governing cell fate determination and differentiation^11–13^. Significantly, 75% of the genome encodes long noncoding RNAs (greater than 200 nucleotides) that have emerged in mammals as global regulators of genome structure. LncRNAs are critically important epigenetic regulators for establishing commitment to cell/organ phenotypes in humans. LncRNA biological functions include modifying enzymes to activate or repress transcription, interactions with epigenetic factors and chromatin. Subsets of nuclear lncRNAs, known as enhancer RNAs (eRNAs), also activate gene expression. Cytoplasmic lncRNAs often have sequence complementarity with other transcripts, which are used to modulate translational control and stability of target RNAs. They can act as competing endogenous RNAs (ceRNAs) or “miRNA sponges”, sequestering specific miRNAs, thereby protecting target mRNA from silencing. In addition, lncRNAs are multifunctional by interacting with numerous components of the gene regulatory machinery (proteins, RNA, DNA).

Identifying and characterizing lncRNAs critical for osteogenesis will extend our understanding of the cellular and molecular mechanisms that regulate bone formation. LncRNAs have been studied as potential biomarkers of aging^14^, osteoporosis^15^ and osteosarcoma^16^. However, there is clearly a gap in knowledge of how lncRNAs mediate epigenetic regulation of skeletal chromatin organization. The nuclear functions of lncRNAs are required to support the commitment of MSCs to a cell lineage and stabilize their phenotype during development, as we recently showed for the lncRNA gene MIR181A1HG^17^. Our goal in this study was to characterize both the functional activities and nuclear mechanisms of LINC01638 in regulating MSC differentiation to the osteoblast lineage. In this study LINC01638 was identified as one of the highly expressed lncRNAs in uncommitted MSCs and required to initiate osteoblast lineage commitment. Our findings directly demonstrate that knockdown (KD) of LINC01638 causes decreased proliferation, senescence, DNA damage, and eventually cell death. Direct examination of chromatin binding through ChIRPseq shows that LINC01638 primarily acts at the chromatin level, targeting genes or modifying the expression of genes related to MSC function and osteogenic differentiation. These results are consistent with a critical function of LINC01638 to protect and maintain the pluripotency of the MSC phenotype. Consequently, LINC01638 supports the requirement of MSCs for tissue development, growth, or repair in different lineages, particularly in bone, for physiologically mediated remodeling.

## Materials and methods

### Cell culture and osteogenic differentiation

Two different sources of human MSCs were used for our studies: Primary human bone marrow MSCs (hMSC) from 5 males were obtained from donors (Texas A&M Health Science Center College of Medicine Institute for Regenerative Medicine at Scott and White). In addition, the immortalized human mesenchymal stromal cell line (hMSC-hTERT20) was generously provided by M. Kassem (University of Southern Denmark, Denmark). Donor hMSCs, immortalized hMSCs-hTERT20 and stably transfected cell lines were maintained in MEMα, nucleosides, no ascorbic acid, supplemented with 16.5% fetal bovine serum, 1% penicillin/streptomycin and 2.0 mM L-glutamine, at 37 °C, 5% CO_2_ and high humidity. Similar to experiments previously described, osteogenic differentiation was induced in complete MEMα supplemented with 280 μM ascorbic acid, 10 mM beta-glycerophosphate and 10 nM dexamethasone^17^. Cells were maintained at 37 °C in a humidified 5% CO_2_ environment, and media was replaced every 2–3 days for the duration of all experiments. Osteoblastic differentiation was assessed by monitoring alkaline phosphatase (ALP) associated staining with Fast Red TR/Naphthol AS-MX^18^. Images were captured with a Leica dissecting microscope (M165FC and Leica digital color camera DFC310FX, Leica Microsystems Inc) and relative activity was quantified using ImageJ software (NIH: https://imagej.nih.gov/). Adipogenic differentiation of MSCs was induced in complete MEMα with 0.5 mM 3-isobutyl-1-methylxanthine (IBMX) (Sigma-Aldrich), 1 μM dexamethasone (Sigma-Aldrich), 1 μM Rosiglitazone (Sigma-Aldrich), 50 μM Indomethacin (Sigma-Aldrich) and 5 μg/mL insulin for a period of 14 days. Adipogenic differentiation was assessed by Oil Red-O staining and images were captured with a Leica dissecting microscope (M165FC).

Primary human MSCs were obtained from the National Institutes of Health-sponsored Center for the Preparation and Distribution of Adult Stem Cells (medicine.tamhsc.edu/irm/msc-distribution.html). The cells were from bone marrow aspirates of normal, healthy donors (donor 2015) with informed consent under Scott & White and Texas A&M Institutional Review Board-approved procedures (ORIP of the NIH, Grant # P40OD011050).

### Cell growth assay

Cells were seeded in six-well plates at an inital density of 5.2 × 10^3^ cells/cm^2^. Cells were trypsinized and counted at specified time points using a Countess 3 Automated Cell Counter (Invitrogen /ThermoFisher Scientific).

### Generation of LINC01638 KD knockdown cells

A stable cell line with a knockdown of LINC01638 was generated using CRISPR interference. The LINC01638 KD hTERT20-hMSC cells were transduced with lentivirus to stably express sgRNA-dCas9-KRAB. LINC01638 KD cells contained multiple sgRNAs targeting the promoter region of LINC01638 (target sequence(s): CACTGTGAACAGTGATGCAG, TCAGGTGAGGAGCTGTTCAT, GTCTGATCACTGAGCAACCC). Control hTERT20-hMSC cell lines were generated by infection with a non-targeting sgRNA (GGGAGGCAACTGAATCATGG). Infected cells were expanded (1–3 passages) and sorted by Fluorescence-activated cell sorting after sgRNA-dCas9-KRAB infection to ensure all cells maintained dCas9-KRAB-mediated GFP expression. The pLV hU6-sgRNA hUbC-dCas9-KRAB-T2a-GFP was a gift from Charles Gersbach (Addgene plasmid # 71237)^19^.

### Flow cytometry analysis

Cells were harvested by trypsinization and fixed in ice-cold 75% ethanol for 30 min at 4 °C. To determine mitotic-associated activity, cells were incubated with Alexa Fluor 647 Rat anti-Histone H3 (pS28) (1:50; BD Biosciences 558609) in a permeabilization buffer for 30 min at room temperature (RT) in the dark as previously reported^17^. For mitotic-association experiments and cell-cycle analysis, cells were stained with propidium iodide (PI/RNase staining buffer, BD Biosciences: 550825) for 15 min at RT. Flow cytometry was performed using an LSRII (BD Biosciences) at the Harry Hood Bassett Flow Cytometry and Cell Sorting Facility at the University of Vermont Larner College of Medicine. Cell cycle analysis by PI incorporation was performed using Flowjo software v10.8 (BD Life Sciences) as well as to determine the percent of H3S28P-positive cells. In addition, fluorescent cell cytometry was used to determine the fraction of cells in S phase and overall DNA content by BrdU (5-Bromo-2′-deoxy-uridine) incorporation using a APC-BrdU Flow Kit with 7-AAD according to manufacturer’s protocol (BD Biosciences).

### Immunostaining and immunofluorescence microscopy

To determine markers of cell proliferation, DNA damage and senescence, cells were assessed by immunostaining followed by immunofluorescent microscopy. Initially cells were grown on coverslips to sub-confluence (50–70% of total surface area). Cells were fixed in 3.7% formaldehyde, washed with PBS, permeabilized in 0.25% Triton X-100 in PBS, then rinsed with PBS-BSA blocking buffer (0.5% bovine serum albumin (BSA) in PBS). Cells were incubated with antigen-specific primary antibodies for 45 min at 37 °C, followed by detection of antigen–antibody complexes using species-specific fluorescently (Alexa Fluor 488, Alexa Fluor 568 or Alexa Fluor 647 (ThermoFisher Scientific)) labeled secondary antibodies and counterstained with DAPI (ThermoFisher Scientific) to identify nuclei. Cells were mounted with ProLong Gold Antifade reagent (ThermoFisher Scientific) on glass slides. Images were either captured using a Zeiss AxioImager2 using Zen2012 software or a Keyence BZ-X800 inverted microscope. To assess DNA double-strand breaks and DNA damage, anti-53BP1 antibody (1:500; Santa Cruz clone H300) and pH2Ax Ser139 antibody (1:500; EMD Millipore cat# 05-636 cloneJBW301) were used. Phosphorylation of histone H3 at the serine 28 residue (H3S28P) antibody (1:500; EMD Millipore cat# 07-145) was used to determine cells in mitosis/ mitotic retention. To examine cellular senescence, an anti-p21 (1:500, BD Pharmingen cat# 556431) and anti-HP1a (1:1000, Millipore cat# MAB3584) antibody was used to stain cells. Image analysis was performed as follows; 53BP1 and pH2Ax specific staining were analyzed utilizing Perkin Elmer’s Volocity 6.3 Software, and p21 and HP1a staining was determined as a proportion of nuclear (PI-stained) area using HALO image analysis platform (Indica labs).

For BrdU assays, BrdU (10 uM) was incorporated for 30 min at 37 °C, then cells were fixed with ethanol/50 mM glycine (pH 2.0) for 20 min at − 20 °C with a BrdU Labeling and Detection Kit according to the manufacturer’s protocol (Sigma-Roche). The extent of BrdU labelling was detected by staining with mouse IgG anti-BrdU primary antibody (1:10), followed by Alexa Fluor 568 goat anti-mouse IgG (1:500) each for 30 min at 37 °C. Cells were counterstained with DAPI for 1 min and mounted with ProLong Gold Antifade (ThermoFisher) and imaged on a Zeiss AxioImager2 equipped Zen2012 software (Zeiss Group AG). Captured image analyses were performed using ImageJ/FIJI.

### RNA in situ hybridization

RNA fluorescence in situ hybridization (RNA FISH) was performed as previously described^17^ using a RNAScope Multiplex Fluorescent Assay (Advanced Cell Diagnostics Bio-Techne, Cat # 571311), according to the manufacturer's protocols with custom probes targeting LINC01638. *Homo sapiens* PPIB and *Escherichia coli* dapB probes were used as positive and negative controls, respectively. RNase A pretreatment was included to confirm probe hybridization to RNA. Images were obtained using a Keyence BZ-X800 microscope, and images were analyzed using the HALO image analysis platform.

### RNA-sequencing

Total RNA was isolated from cell pellets using Trizol and further purified using the Direct-zol RNA Kit (Zymo Research). Purified RNA was treated with DNAse I and quantified using Qubit RNA broad range (BR) reagents on a Qubit 4 fluorometer (Invitrogen/ThermoFisher Scientific). RNA quality was then assessed using the RNA 6000 Nano Kit with the Agilent 2100 Bioanalyzer (Agilent Technologies). RNA-Seq libraries were built with the SMARTer Stranded Total RNA Sample Prep Kit—Hi Mammalian Kit (Takara) or TruSeq Stranded Total RNA Library Prep Kit with Ribo-Zero Gold (Illumina) according to the manufacturer’s protocol. Resultant libraries were assessed by Agilent 2000 Bioanalyzer using a DNA HS chip (Agilent). Libraries were single-end sequenced on a HiSeq-1500 at the Vermont Integrative Genomics Resource Massively Parallel Sequencing Facility. Base calls and sequence reads were generated by bcl2fastq software (version 1.8.4, Illumina).

### Bioinformatics Analysis

To analyze RNAseq and ChIRPseq data the following analysis strategy (described previously^17^) was used. Initial quality control of fastq data was assessed using FastQC^20^. Reads passing QC were aligned to reference genome (hg38) using STAR aligner^21^ with GENCODE annotation v42^22^, and alignments were quantified using FeatureCounts/HTSeq-counts^23^. A minimum cutoff value of 0.1 RPKM was used to assess significant gene expression and then differential expression analysis was performed using DESeq2^24^. The cutoff for significant fold change for differential gene expression analyses was > 1.5, adjusted *p* value < 0.05. Cells at two differentiation stages (day 7 and 14) were compared with undifferentiated cells (day 0) as well as to each other within each cell type (Control and LINC01638 KD). In addition, expression of LINC01638 was compared to normalized expression counts from normal tissues Genotype-Tissue Expression (GTEx) Portal and in cancer subtypes using data from the Cancer Genome Atlas Program (TCGA). Clustering analysis of row normalized differentially expressed gene count data was performed for each group using K-means clustering and visualized using SeqSetVis^25^. mRNA groups with similar expression patterns were merged, and Gene Ontology (GO) annotation and Gene Set Enrichment Analyses (GSEA) of gene sets were performed using Gene Ontology of mSigDB (Broad Institute)^26,27^. GO Term enrichment was considered significant for all terms with *P* < 0.05. GO terms were consolidated using REVIGO^28^.

### ChIRP-qPCR and ChIRP-Seq

ChIRP experiments were performed following the method described in Chu et al.^29^ and described previously^17^ with certain modifications. Briefly, 4 × 10^8^ cell pellets were crosslinked by resuspension in freshly prepared 1% glutaraldehyde followed by cross-linking with 3% formaldehyde. For qPCR analysis of ChIRP libraries, PCR primers were designed to specific genomic regions corresponding to gene exons or regulatory regions (e.g., enhancers, promoters) as previously reported^17^ and qPCR was carried out in reactions using 10 pg of library DNA using QuantiFast SYBR Green qPCR kit (Qiagen) using standard cycle parameters on Viia7 Real-time PCR thermocycler (ThermoFisher). Relative enrichment was calculated by normalizing Ct values to recovered input DNA (percent input) and then calculating fold enrichment compared to the control lncRNA (MALAT1).

### Statistical analyses

Statistical analyses were performed using GraphPad Prism v8.4.3 and/or R. For individual experiments statistical tests are described in figure legends.

### Ethics approval statement

The use of cells obtained from human subjects was approved by the Texas A&M College of Medicine-Scott and White Medical Center Institutional Review Board and by the University of Vermont Institutional Review Board (study 15-629).

### Approval for human studies

For experiments involving human participants (specifically human primary MSCs) informed consent was obtained from all subjects and/or their legal guardians by the National Institutes of Health-sponsored Center for the Preparation and Distribution of Adult Stem Cells at Texas A&M College of Medicine-Scott and White Medical Center. All methods involving human tissues were performed in accordance with the relevant guidelines and regulations from the Texas A&M Institutional Review Board or by the University of Vermont Institutional Review Board (study 15-629).

### Accession of data

All datasets have been deposited in the Gene Expression Omnibus (GEO). Datasets used in this study have been deposited in the Gene Expression Omnibus (GEO) under accession numbers GSE227512, GSE185951, GSE184087 and GSE183931.

## Results

### LINC01638 expression is associated with MSCs

**Figure 1:**
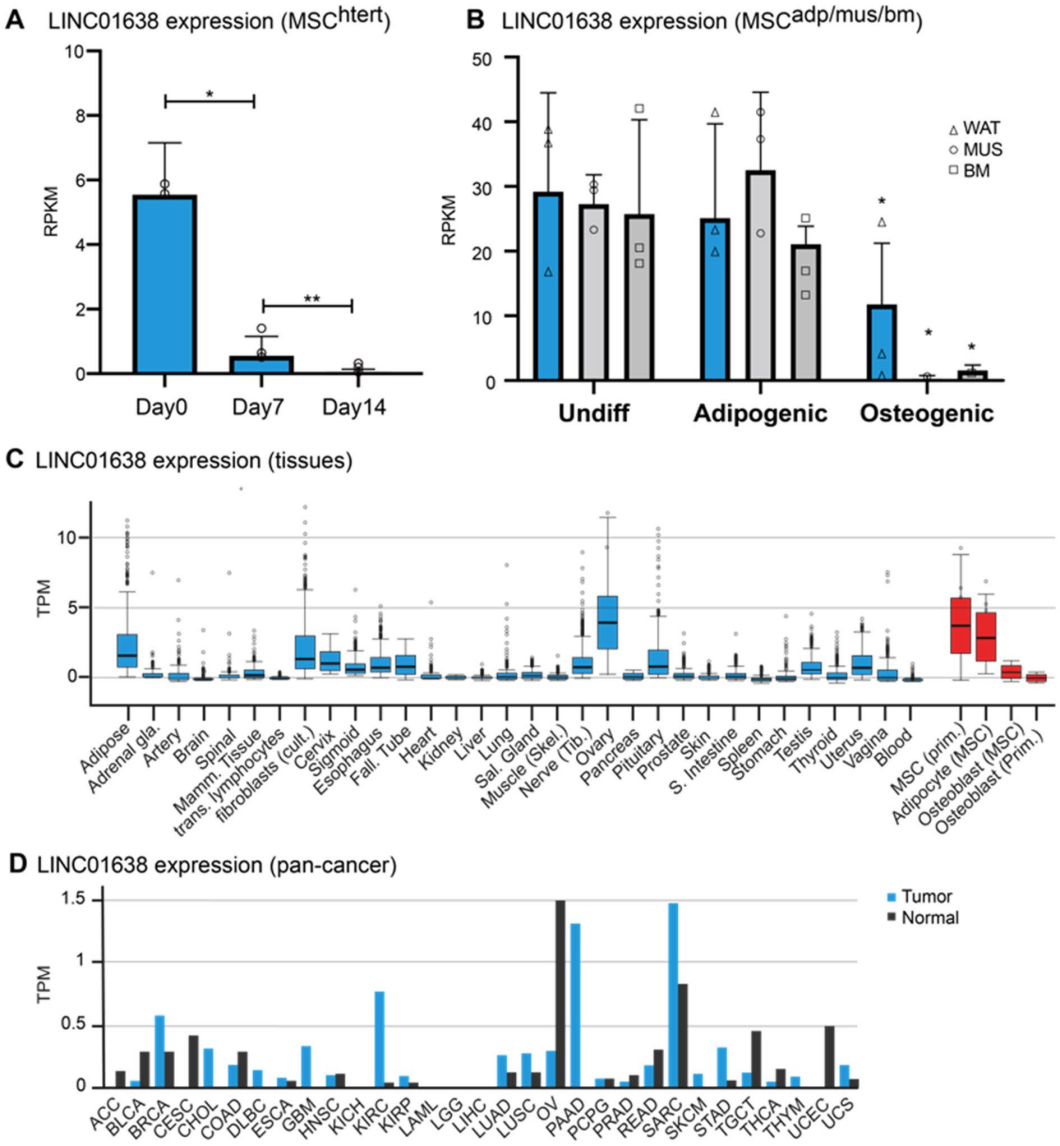
LINC01638 is expressed primarily in undifferentiated MSCs. (**A**) LINC01638 expression in MSC (hTERT20) undergoing osteogenic differentiation. LINC01638 expression significantly decreased (*p > 0.01, **p > 0.05) during osteogenic differentiation. (**B**) LINC01638 expression in MSCs derived from white adipose tissue (WAT), muscle (MUS) or normal bone marrow (BM) undergoing adipogenic or osteogenic differentiation. LINC01638 was significantly decreased (*p > 0.01) in all MSC types undergoing osteogenic differentiation. (**C**) LINC01638 expression across human tissues as determined by RNAseq (GTex). The lncRNA is not detected in the majority of examined tissues except in articular chondrocytes and low levels in testis and mammary samples, whereas expression was high in ovary tissue and undifferentiated MSCs. (**D**) Expression of LINC01638 in TCGA tumor subtypes.

We had previously characterized global long non-coding RNA (lncRNA) expression in human MSCs undergoing osteogenesis^17^. We identified LINC01638 as being highly expressed in undifferentiated MSCs and significantly decreased expression during osteogenic differentiation (Fig. 1A). This expression was reduced during temporal expression as levels of LINC01638 were significantly decreased at day 7 and negligible at day 14 of osteogenic differentiation (Fig. 1A). The expression of LINC01638 was similar in MSCs isolated from adipose-, muscle- or bone marrow-derived MSCs^30^ (Fig. 1B). Expression of LINC01638 was not changed during adipogenic differentiation; however it was significantly decreased during osteogenic differentiation in all three cell types (Fig. 1B). We defined tissue specific expression of LINC01638 (GteX) and determined it was restricted largely to MSC-derived linages, although there is a notable high level of LINC01638 expression in mature ovary tissues (Fig. 1C). Additionally, LINC01638 expression is elevated in several cancer types and is specifically elevated in mesenchymal-derived sarcomas compared to normal control tissue (Fig. 1D).

### LINC01638 knockdown is associated with senescence, quiescence and DNA damage

**Figure 2:**
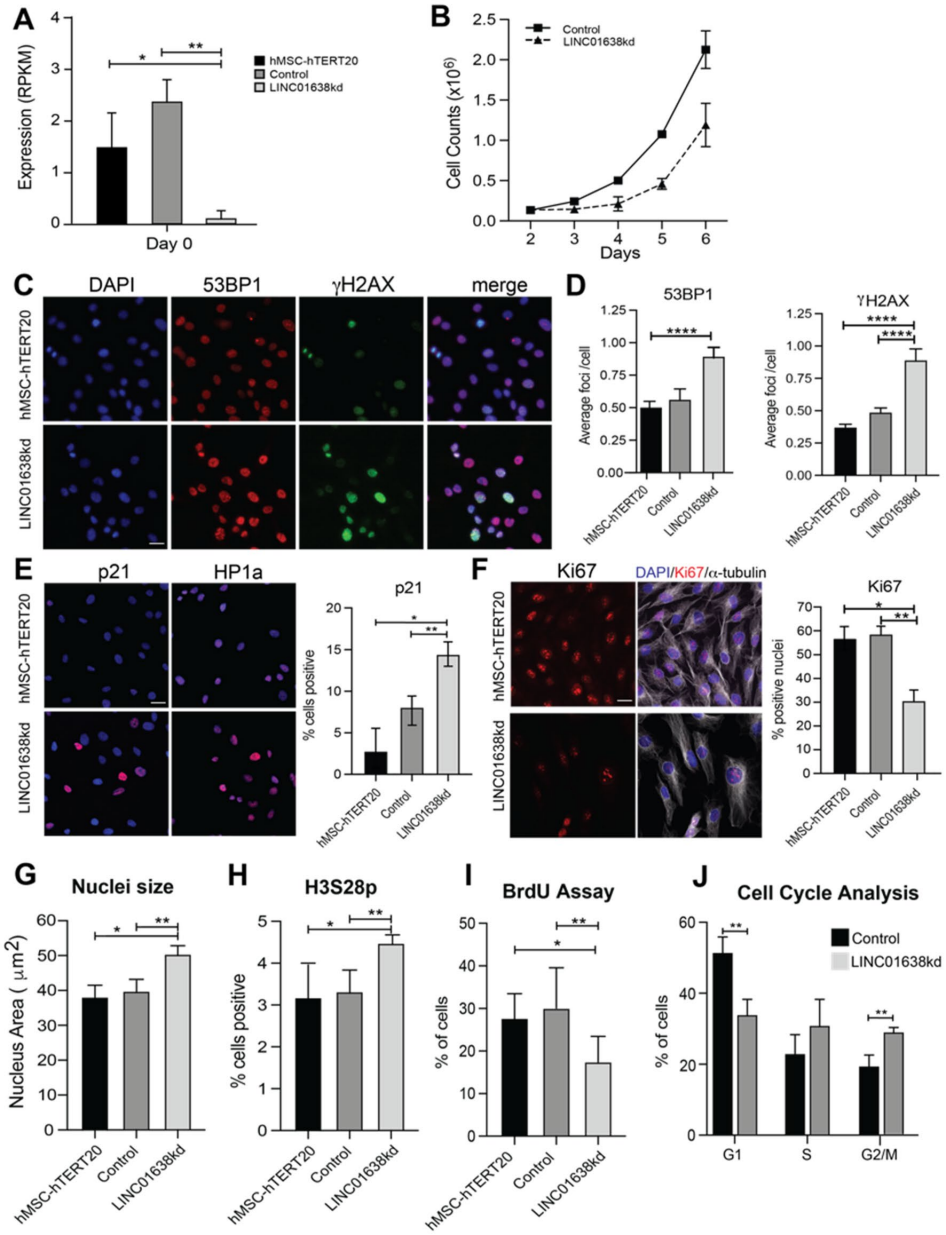
LINC01638 KD reduces cell proliferation and increases markers of cellular senescence. (**A**) Expression of LINC01638 upon CRISPRi knockdown, relative to Control and MSCs (*p < 0.05 vs hTERT). (**B**) Cell growth reduction in response to LINC01638 KD versus Control. (**C**) Immunofluorescence images of DAPI, increased 53BP1 and γH2AX in LINC01638 KD cells (Scale bar = 20 µM). (**D**) For each cell line 53BP1 or γH2AX foci were counted from > 600 cells from 2 independent experiments (****p < 0.0001). (**E**) Immunofluorescence images of p21 and H1Pa immunostaining staining [counterstained with DAPI (blue)]. P21 positive nuclei were counted and expressed as % of cells (> 900 cells quantified from n = 2 experiments) (*p < 0.001 vs hTERT, ** vs control). (**F**) Immunofluorescence images of Ki67 (red), DAPI (blue) and α-tubulin staining (gray scale). Quantification of Ki67 intensity for > 1000 cells by HALO from n = 2 experiments (*p < 0.0001 vs hTERT, ** vs control). (**G**) Nuclei area calculated from > 1000 cells by HALO from n = 2 experiments (*p < 0.0001 vs hTERT, ** vs control). (**H**) H3S28 phosphorylation; and (**I**) BrdU positive nuclei were counted (> 1000 cells quantified from n = 2 experiments) (*p < 0.001 vs hTERT, ** vs control). (**J**) Cell cycle analysis quantifying the number (percentage) of cells in respective cell cycle phase.

Knockdown of LINC01638 showed decreased expression on day 0 (Fig. 2A) and a decrease in cell proliferation (Fig. 2B). Gamma H2AX and 53BP1 are markers for double strand DNA breaks (DNA damage). 53BP1 is diffused in the nucleoplasm in undamaged cells and upon damage or double stranded breaks, cells show punctate focal staining patterns. There seems to be a higher population of damaged cells in the LINC01638 knockdown in comparison to the control hMSCs. Both gamma H2AX, 53BP1 (sensors for DNA damage response) (Fig. 2C, D) as well as P21 (an inhibitor of cell proliferation) were upregulated upon LINC01638 knockdown (Fig. 2E). These markers for double-strand DNA breaks (DNA damage) and proliferation each show a higher frequency of damaged cells upon LINC01638 knockdown in comparison to the control hMSC (Fig. 2C–E). These results are consistent with LINC01638 KD blocking differentiation. The senescence marker HP1alpha is upregulated in LINC01638 KD cells (Fig. 2E). In addition, the proliferation marker Ki67 was decreased in LINC01638 KD cells (Fig. 2F). One of the hallmarks of senescence is alteration of the cytoskeleton^31^. LINC01638 KD cells displayed altered cellular morphology and α-tubulin organization (Fig. 2F) compared to controls. LINC01638 KD increased the nuclear size and irregularity which are indicators of damaged cells (Fig. 2G). Cell cycle analysis by BrdU incorporation and flow cytometry confirmed a significant decrease in G1 phase cells with an increase in G2/M phase cells after LINC01638 KD; however, there was no significant increase in the number of cells in S phase (Fig. 2I, J). LINC01638 is clearly functioning to maintain the cell stability and assure the proliferation of MSCs to be used for the differentiation of MSCs to distinct lineages when required for tissue development or maintaining bone homeostasis of osteoblasts (e.g., in times of bone turnover). Approximately 35.6% and 33.9% of LINC01638 cells have staining in γH2Ax and 53BP1 respectively, in comparison to 27.7% and 28.9% of hMSC cells. Pixel counts in 53BP1 and γH2Ax quantify the accumulation of damage-associated markers. A higher pixel count can be associated with more DNA damage. More DNA damage is an indicator cells blocked in G0_._ As shown in Fig. 2E, LINC01638 had higher pixilation in 53BP1 and γH2Ax in comparison to the control cell line. Pixel counts more than doubled in the LINC01638 cell line in both DNA damage markers in comparison to the control cell line. Counts rose from 64.4 (hMSC) to 164.2 (LINC01638) in the 53BP1 marker in comparison to 17.8 (hMSC) to 38.8 (LINC01638) in γH2Ax.

### LINC01638 expression is required for osteogenic lineage commitment

We then performed knockdown experiments to validate if LINC01638 had a role in regulating MSC commitment. We determined that upon LINC01638 KD, hMSCs exhibited a decrease in proliferation. We investigated if the decrease in proliferation was associated with DNA damage or genomic stability by γH2Ax/p21/53BP1 (Fig. 2). We then knocked down LINC01638 during osteogenic differentiation and found that ALP expression was significantly affected, as well as osteogenesis-associated genes and several other gene pathways. Although osteogenesis was specifically affected, the majority of affected genes (LINC01638 KD) were associated with DNA damage, cell cycle, and chromosomal organization categories.

**Figure 3:**
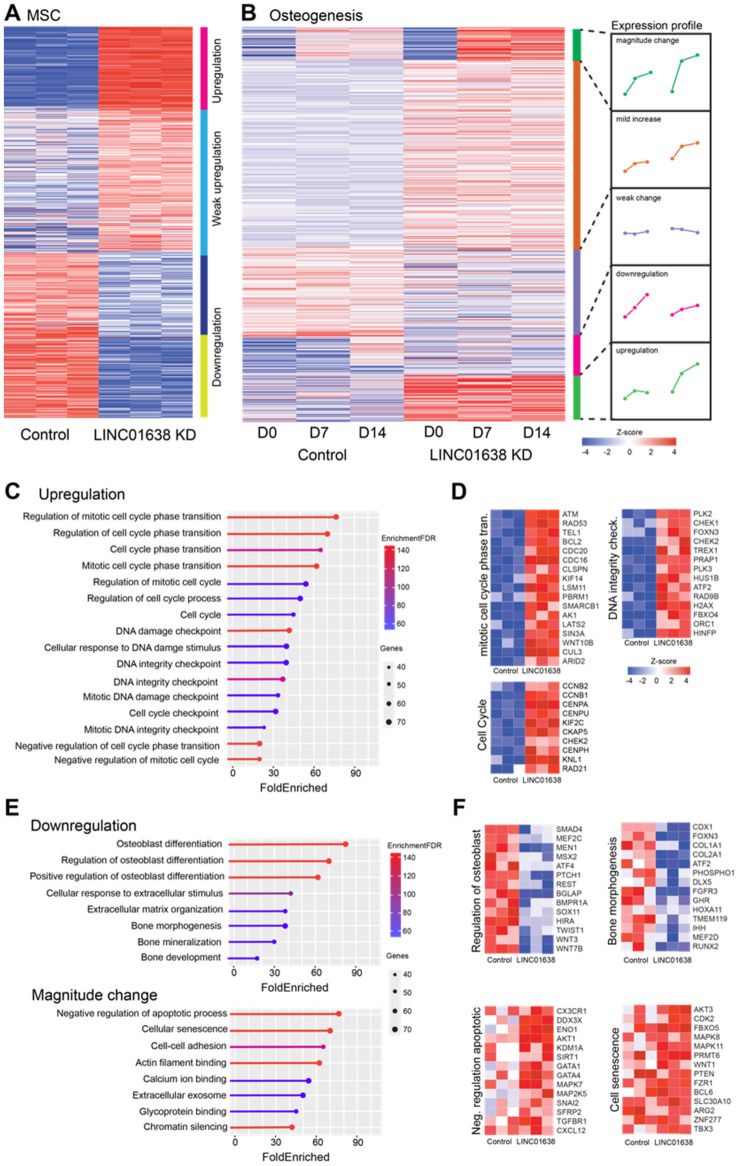
LINC01638 KD results in widespread changes in transcriptional activity. (**A**) Heatmap and hierarchical clustering of 1365 DE mRNAs in control (hMSC-hTERT20) versus LINC01638 KD. (**B**) Hierarchical clustering of 2054 DE genes in control versus LINC01638 KD that change during temporal stages (Day 0, Day 7, Day 14) of osteogenic differentiation. Clusters were segmented into expression profiles (magnitude change, mild increase, weak change, downregulated, upregulated). Values displayed are row-normalized z-score. (**C**, **E**) Gene-set enrichment analysis of genes identified by hierarchical clustering. Plots display ontology category, fold enrichment (x axis scale), number of genes (dot size) and enrichment FDR (line color). (**D**, **F**) Heatmaps of gene expression of representative genes from selected ontological categories. Gene expression values are plotted as row-normalized z-scores. Heatmaps were generated by R [(4.2.2) using packages ggplot2 (3.4.4) and pheatmap (1.0.12)].

Hierarchical clustering of the differentially expressed mRNAs in naïve (untreated) MSCs versus LINC01638 KD MSCs (Fig. 3A) demonstrated 356 genes that were differentially upregulated (in LINC01638 KD), 708 genes that were significantly differentially expressed however classified as weak upregulated or weak downregulated, and 301 genes that were classified as downregulated (Supplementary table 1). Focusing on the overall role of LINC01638 KD in MSC commitment to osteogenesis, we further examined the effect of knockdown upon gene expression during differentiation.

Hierarchical clustering of differentially expressed mRNAs in osteogenic media-treated MSCs was performed to evaluate gene expression changes during distinct timepoints during osteogenesis. Differential expression analysis demonstrated that 2054 genes were affected by LINC01638 KD (Fig. 3B). Hierarchical clustering was used to segregate genes into patterns of temporal expression which include genes that increased in relative magnitude of expression (magnitude change, 154 genes), significant but mild change in expression, weak or no change, downregulated (256 genes), or upregulated (208 genes).

There was substantial overlap between genes upregulated in naïve LINC01638 KD and LINC01638 KD MSCs undergoing osteogenesis. Examination of the combined 378 upregulated protein-coding genes that were upregulated upon LINC01638 knockdown by gene ontology analysis demonstrated that upregulated genes were significantly associated with ontologies and categories associated with regulation of cell cycle and specifically regulation of mitotic cell cycle (Fig. 3C). The top ranked ontology category was regulation of mitotic cell cycle phase transition which was significantly enriched over a weighted background gene list. Genes associated with this ontological category include ATM, BCL2, CDC16, WNT10A, as well as several other genes regulating M/G2 cell cycle progression that were all significantly upregulated in LINC01638 KD cells (Fig. 3D). In addition, genes linked to cell cycle and DNA integrity checkpoint ontologies were also upregulated in LINC01638 KD cells (Fig. 3D). These data support the observed decrease in cellular proliferation and subsequent increase in DNA damage and senescence markers observed in LINC01638 KD cells.

Focusing on the overall role of LINC01638 KD in MSC commitment to osteogenesis, we further examined genes that were downregulated during osteogenesis. We evaluated by gene ontology analysis, and as expected, gene ontology categories associated with osteogenesis were the top ranked and most significantly associated ontologies (Fig. 3E). Expression of several genes were associated with extracellular matrix organization, regulation of osteoblast differentiation, and bone morphogenesis (Fig. 3F). Several canonical bone genes were included in this cluster of downregulated genes including SMAD4, ATF4, BMPR1A, COL1A1, IHH, and RUNX2 (Fig. 3E). These data would suggest that LINC01638 KD results in decreases in multiple genes required for osteogenesis. Interestingly, hierarchical clustering demonstrated a pattern of genes that changed in magnitude during osteogenesis that upon gene ontology analysis showed significant association with diverse ontology categories including negative regulation of apoptotic process, cellular senescence, and chromatin silencing (Fig. 3E). Several genes in the cellular senescence category including FBXO5, WNT1, FZR1, and several others were increased on Day 7 and Day 14 of osteogenic differentiation (Fig. 3F). This would suggest that the increased expression of these specific genes may contribute to increased senescence that prevent full commitment to the osteogenic lineage in MSCs.

### LINC01638 has restricted expression in hMSCs for maintaining normal bone tissue development

**Figure 4:**
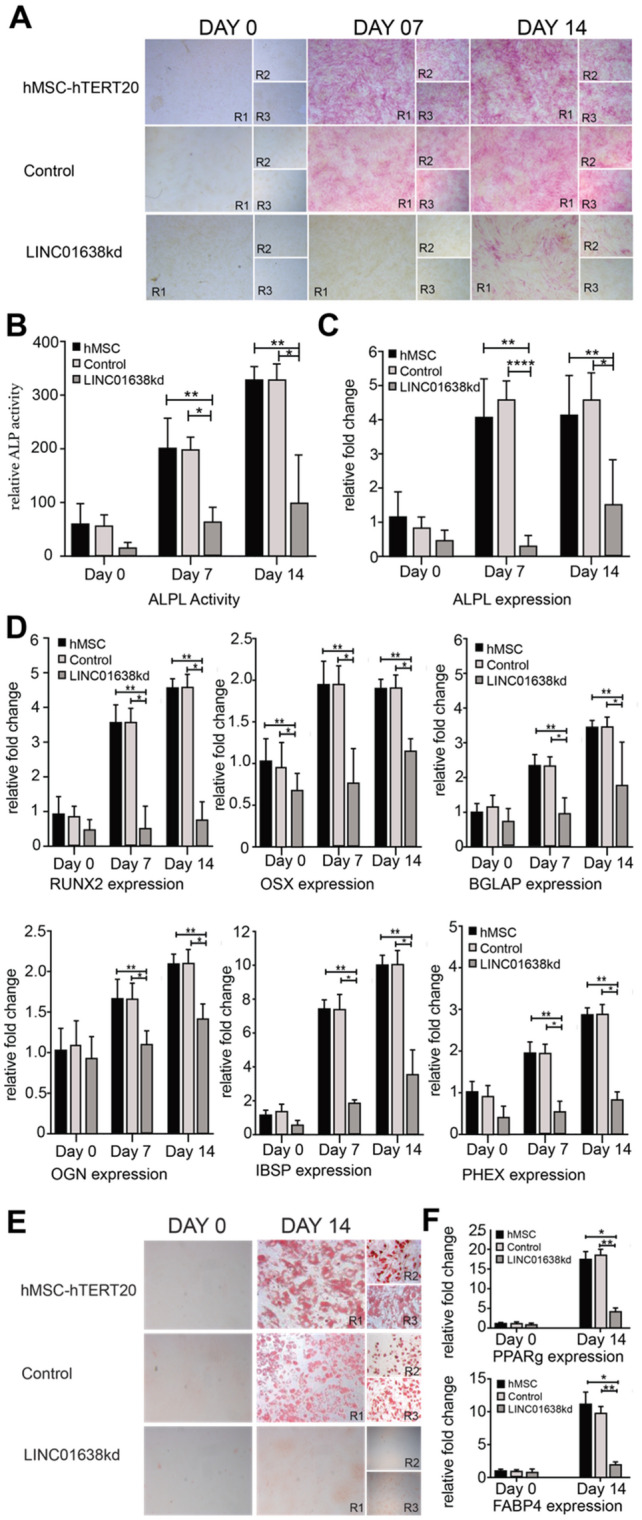
LINC01638 is required to initiate differentiation to the osteogenic lineage. (**A**) Alkaline phosphatase staining in MSCs (hTERT20, Control (CRISPR), or LINC01638 KD) undergoing osteogenic differentiation at 0, 7, or 14 days. Experiments were performed in triplicate and each represented replicate is indicated (R1-3). (**B**) ALP activity was measured and displayed as relative fluorescent activity units. (**C**) ALPL expression measured by qPCR analysis. Measurements are displayed as relative fold expression (normalized to hMSC Day 0 values). Values are measured from multiple replicates (n = 3) and values displayed as mean +/− SD. Statistical significance was determined by one way ANOVA (**p > 0.01 (compared to hTERT20), *p > 0.01 [compared to Control (CRISPR)]. (**D**) Osteoblast related gene expression (RUNX2, OSX, OCN, OGN, IBSP, and PHEX) measured by qPCR analysis. Measurements are displayed as relative fold expression (normalized to hMSC Day 0 values). (**E**) Oil Red-O staining of MSCs unsergoing adipogenic differentiation. (**F**) Adipogenic-related gene expression 9PPArg, FABP4) measured by qPCR analysis. Measurements are displayed as relative fold expression (normalized to hMSC Day 0 values). Values are measured from multiple replicates (n = 3) and values displayed as mean ± SD. Statistical significance was determined by one way ANOVA (**p > 0.01 (compared to hTERT20), *p > 0.01 [compared to Control (CRISPR)].

Using ALPL as a screening for the extent of osteogenesis during induced differentiation from hMSCs, we observed slow growth rate and low levels compared to the human Tert-immortalized cells (Fig. 4A). These levels were confirmed by RNAseq, ALP expression, and qPCR (Fig. 4B). We also found that LINC01638 knockdown further decreased ALPL expression, and completely inhibited osteogenic differentiation. LINC01638 was depleted by CRISPRi using dCas9-KRAB, and the hMSCs were then treated with osteogenic media for 14 days with low ALP activity (Fig. 4A, B) and expression (Fig. 4C). In addition, several other bone-related genes were significantly decreased at 7 and/or 14 days of differentiation in LINC01638 KD cells. These genes include canonical osteogenic transcription factors: RUNX2 and OSX, as well as osteoblast markers BGLAP, OGN, IBSP, and PHEX (Fig. 4D), suggesting that the entire osteogenic gene program was affected by the removal of LINC01638. As LINC01638 knockdown was associated with impaired commitment to the osteogenic lineage, we examined whether adipogenic commitment was also impaired. LINC01638 knockdown resulted in a clear reduction of lipid vesicle formation by Oil Red-O staining (Fig. 4E) and adipocyte-related gene expression (PPARg, FABP4) compared to controls (Fig. 4F). These results suggest that the role of LINC01638 may be critical to MSC commitment to the osteogenic as well as the adipogenic lineages.

### LINC01638 interacts with chromatin during MSC commitment

**Figure 5:**
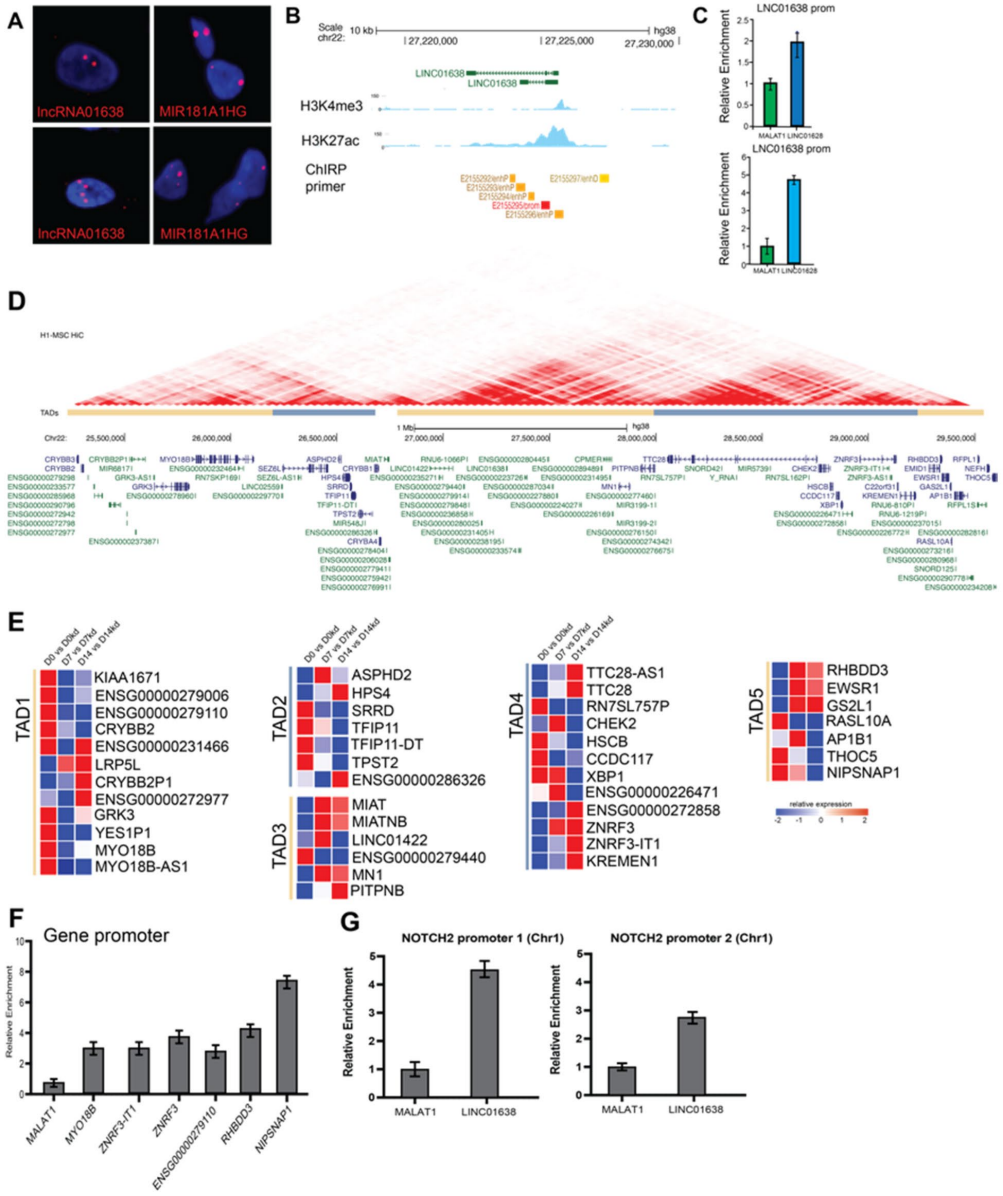
LINC01638 is located in the nucleus and interacts directly with chromatin at specific genomic regions. (**A**) RNA-FISH demonstrating discrete focal association of LINC01638 in the nucleus of hMSCs. (**B**) UCSC genome browser ideogram depicting LINC01638 locus on chromosome 22 with associated regulatory regions and specific histone modifications (H3K4me3 and H3K27ac); ChIPseq tracks from MSCs are depicted demonstrating active regulatory regions around assigned gene promoters (blue); (**C**) Quantitative PCR of ChIRP DNA from LINC01638-associated probes. Relative enrichment compared to control (MALAT1) demonstrates that LINC01638 is specifically associated with the LINC01638 gene locus and regulatory regions. (**D**) UCSC Genome Browser view of gene cluster on Chr22 proximal to the LINC01638 locus with H1-MSC Hi-C data demonstrating interaction frequency and TADs. (blue, yellow) (**E**) Gene expression changes in genes proximal to the LINC01638 gene locus on Chr22. Expression is displayed as fold difference relative to control. (**F**) ChIRP analysis of LINC01638 binding to chromatin sites (gene promoters) proximal to LINC01638 locus on Chr22. Relative enrichment was determined by qPCR and plotted as mean ± SD. (**G**) ChIRP analysis of LINC01638 binding to the NOTCH2 promoter (on Chr1).

Our findings indicated that LINC01638 may have a direct role in chromosomal organization; we looked to see if LINC01638 was associated with chromatin by RNA-FISH (Fig. 5A). We observed LINC01638 at discrete sites of focal association in the nucleus suggesting a specific nuclear role similar to a previously identified nuclear-associated LncRNA (Mir181A1HG)^17^. The direct association of LINC01638 on chromosomes indicated that LINC01638 may work to directly regulate gene expression or chromatin in the nucleus of hMSCs. We also performed ChIRP to identify regions where LINC01638 is associated. ChIRP analysis demonstrated that LINC01638 was enriched on its own genomic locus on Chr22 (Fig. 5B, C). Interestingly, the LINC01638 genomic locus on Chr22 is associated with a lncRNA-rich region, encoding several lncRNAs (Fig. 5D) within this region, as well as multiple protein-coding genes. We then asked if genes within this region showed co-expression (co-ordinated) patterns upon LINC01638 KD. Several genes in this region demonstrated differential expression upon LINC01638 KD, with the majority of genes being upregulated (Fig. 5E). Several of these genes were linked with osteogenesis or GWAS for bone mineral density (e.g., ZNF3, KREMEN1), several were cell cycle-related genes (e.g., CHEK2), and several were lncRNAs. ChIRP analysis of gene promoters proximal to the LINC01638 locus on Chr22 demonstrated that LINC01638 was bound to the promoter regions of several genes (MYO18B, ZRNF3, RHBDD3, and NIPSNAP1) (Fig. 5F). This suggests that LINC01638 directly regulates genes close to its own locus. We sought to determine if there was an association of LINC01638 and NOTCH genes in MSCs given the role of NOTCH in osteogenesis. We performed ChIRP-qPCR for NOTCH1 and NOTCH2 promoters and observed enrichment of LINC01638 (Fig. 5G) at the gene promoter which corresponded with a significant decrease in NOTCH expression (Fig. 5G). This would suggest that LINC01638 negatively regulates NOTCH2 directly, as well as several other genes associated with osteogenesis, leading to the overall block in MSC commitment and reduction in osteogenic activity.

## Discussion

Recent findings have demonstrated that lncRNAs exert a level of epigenetic control to promote and define cellular lineages, regulate gene expression, and support genomic/chromosomal architectural organization through their direct interactions across the genome^4,33–36^. Our characterization of LINC01638, as one of the highest expressed lncRNAs in hMSCs, reveals novel insights related to osteogenesis. Multiple experimental approaches for interrogating its unique properties provide options for hMSC commitment to specific lineages controlled by LINC01638. A key finding of our studies is the essential role of LINC01638 to preserve hMSC competency to regulate distinct lineages when required for different tissues, including bone.

Examining the tissue-specific expression of LINC01638 indicated it was expressed at low levels in most tissues (GTEx Portal), although there was a similar level of expression in ovarian tissues (compared to MSCs). Upon initiation of hMSC commitment to the osteogenic lineage, we observed a striking down-regulation of LINC01638. We tested whether the knockdown of LINC01638 would reverse the inhibition and allow osteogenesis to proceed through stages of osteoblast differentiation. However, the knockdown of LINC01638 resulted in a proliferation decrease, cell senescence, and cell death compared to the hMSC control. We determined that the decrease in proliferation was associated with DNA damage and genomic instability mediated by γH2AX, p21, and 53BP1. Thus, we identified that the most significant role of LINC01638 is protecting a pool of proliferative hMSCs to support lineage commitment to a specific organ/cell phenotype. Our findings confirm that LINC01638 is stringently regulated to assure the ability of hMSCs to support tissue development and remodeling that is required for skeletal homeostasis.

We demonstrated that alteration of LINC01638 expression in hMSCs undergoing osteogenic commitment resulted in differential expression of genes directly upstream and downstream of the LINC01638 locus on chromosome 22 (Chr22). Genes related to cell cycle control, DNA damage, and osteogenesis (GWAS, Bone Mineral Density (BMD)-associated) were identified in this cluster. The large cluster on Chr22 where this lncRNA is encoded is associated with bone-related genes, both protein and noncoding. The knockdown studies identified the up- and down-regulation of LINC01638 proximal genes, including genes critical for bone formation, such as Kremen1, that function to regulate bone formation via attenuating Wnt signaling in the developing limb to allow normal limb patterning. Kremen1 is known to be highly expressed in mature bone^37^ and directly involved in maintaining bone density^37,38^. ZNRF3 is another proximal gene that is involved in Wnt signaling through modulation of LRP4/5/6 activity^39^. This would suggest that LINC01638 regulates genes proximal to its own locus that significantly impact bone formation and cell fate. Of importance, the Chr22 LINC01638 locus was significantly enriched in other novel lncRNAs, which are of unknown function. In addition, several genes are differentially expressed and related to osteogenesis, affected by LINC01638 KD. It is interesting that several lncRNAs in the same genomic location are similarly regulated compared to LINC01638, and this warrants further investigation.

We further pursued the direct chromatin interactions with LINC01638 by ChIRP studies (ChIRP-qPCR) which confirmed chromatin binding on Chr22, identifying an enriched region of interaction. From our ChIRP PCR studies, we again identified that the KREMEN1, ZNRF3, and NOTCH genes are bound by LINC01638 suggesting a direct regulation of gene expression through genomic interactions, presumably through recruitment of transcriptional mediators, chromatin modifying complexes, or stabilization of transcriptional loops or domains, as has been demonstrated for other lncRNAs^40^. Importantly, the KD of LINC01638 results in upregulation of Kremen 1 and ZNRF3 that both inhibit Wnt signaling and in turn bone formation. Taken together, given the integral role of these genes in osteogenesis and bone maintenance, it is clear that LINC01638 is an important epigenetic regulator for maintaining MSC integrity and can function when required for bone renewal.

We recognize that the mechanisms contributing to bone lineage fate are not fully understood. Additional evidence that LINC01638 directly regulates osteogenesis is by its interaction with the NOTCH2 gene located on Chr1. Notch signaling has a major role in the commitment of mesenchymal cells to the osteoblastic lineage. Notch expression in osteoblast precursors regulates femoral microarchitecture^41–43^. Our discovery provides a novel and key pathway mediated by LINC01638 in regulating bone tissue. How these important functions of LINC01638 in regulating KREMEN, ZNRF3, and NOTCH through recruitment of co-regulators during osteogenesis remain to be further studied. Nonetheless, our findings reveal a novel dimension of bone regulation by LINC01638.

LINC01638 functionally contributes to regulation of the balance between osteogenesis and adipogenesis. Studies have shown that fat-induction factors inhibit osteogenesis, and conversely, bone-inducing factors impair adipogenesis^44,45^. The commitment of MSCs in forming fat or bone are related to pathological conditions, such as early osteoporosis in bone and/or aging-associated adipogenesis. It has been well documented that secreted factors in the bone marrow microenvironment mediate a cross-talk of lineage secreted factors between bone and fat. Numerous recent studies have demonstrated that miRNAs and lncRNAs can regulate fat and bone tissue depending on the required condition. For example, secreted frizzled-related protein sFRP-1 (osteogenic related) and Dlk1/Pref-1 (preadipocyte factor1) contribute to the regulatory effects of both adipogenesis and osteogenesis^44,46,47^. Our finding that LINC01638 is highly expressed in undifferentiated MSCs was somewhat surprising and could signify a context-dependent role for LINC01638 in MSC commitment to a specific lineage.

LINC01638 is expressed at low levels in normal tissues, and numerous studies have reported its expression in multiple cancers^7,48^. Inhibition of LINC01638 reduces tumor growth in cancers, including prostate and breast, both of which metastasize to bone^48,49^. In addition, higher expression of LINC01638 has been suggested to be a poor prognostic marker for triple-negative breast cancer^50^ and induced overexpression of LINC01638 in pancreatic ductal adenocarcinoma. Induced overexpression of LINC01638 in pancreatic ductal adenocarcinoma promoted cell migration and invasion through epithelial-to-mesenchymal transition (EMT)-like mechanisms^51^. It should also be noted that in triple negative breast cancer (TNBC) cells, LINC01638 was found to maintain mesenchymal traits of TNBC cells, including promoting the expression of EMT signature genes and a cancer stem cell-like state^52^. In addition, LINC01638 expression in papillary thyroid carcinoma cells has been demonstrated to regulate cell proliferation via interactions and modulation of the Wnt/beta-catenin pathway and activation of Axin2^53^. These studies, along with the results presented here, would suggest that LINC01638 is a strong determinate of MSC identity, driving mesenchymal gene expression in transformed cancer cells. This lncRNA may act as a critical gene in normal MSCs regulating proliferation, protection from DNA damage and cell commitment to the osteogenic lineage. Further, our ChIRP studies reveal chromatin-associated genes that contribute to the stabilization and reorganization of chromatin interactions by LINC01638 that may be important in cancer cell survival and progression.

Taken together, it is clear from our findings that LINC01638 has unique properties to protect the self-renewing MSC population and to support growth and/or repair of bone tissue that is in a constant state of turnover. Therefore, it will be informative to obtain clinical data necessary to uncover the deeper mechanisms by which LINC01638 can mediate physiological control of bone structure and function.

### Supplementary Information

Supplementary Information.
